# Barriers to implementation of emergency obstetric and neonatal care in rural Pakistan

**DOI:** 10.1371/journal.pone.0224161

**Published:** 2019-11-05

**Authors:** Sajid Haider, Rana Farhan Ali, Munir Ahmed, Asad Afzal Humayon, Muhammad Sajjad, Jamil Ahmad

**Affiliations:** 1 Department of Management Sciences, COMSATS University Islamabad, Vehari, Pakistan; 2 Health and Nutrition Supervisor, Chishtian, District Health Office, Bahawalnagar, Pakistan; University of Brighton, UNITED KINGDOM

## Abstract

**Background:**

Recognizing the need for improving maternal and newborn care, the Punjab public health department (Pakistan) launched emergency obstetric neonatal care (EmONC) services under WHO guideline. Unfortunately, the program implementation is facing some serious problems. The purpose of this study was to identify barriers to implementation of EmONC in district Bahawalnagar (Pakistan).

**Methods:**

This study used sequential exploratory design. Specifically, a qualitative study was conducted to identify barriers to EmONC. Subsequently, the relative importance of these barriers was determined in a quantitative study. Participants were health service providers involved in 24-hours basic EmONC services in the basic health units of district Bahawalnagar (Pakistan). Qualitative data were gathered by interviewing the participants using key informant guide. Quantitative data were collected in a rank order survey of the same participants. The methodological quality was assessed using mixed methods appraisal tool (MMAT) version 2011.

**Results:**

The results indicate that lack of teamwork, conflict management, communication, and improper power distribution are important interpersonal barriers. The significant organizational-level barriers include job insecurity, lack of organizational culture, human resource deployment issues, and lack of role clarity. Lack of target management, lack of resource availability, house job requirement, and dual practice issues were identified as major system-level barriers.

**Conclusion:**

Barriers to EmONC implementation must be addressed for improving maternal and neonatal care in district Bahawalnagar.

## Introduction

Maternal mortality is a big global issue with 216 deaths per 100,000 live births [1]. Pregnancy and childbirth related problems are causing 830 maternal deaths around the world per day[2]. It is worth noticing that in 2015 “roughly 303,000 women died during and following pregnancy and childbirth” [2]. The situation is even alarming in developing countries where 99% of the global maternal deaths occur [1, 3]. High rates are especially linked to Africa, South East Asia and South Asia [4]. Despite the fact that the Millennium Development Goals (MDGs) provided the global community with an exciting opportunity to improve women health and reduced maternal mortality [5], they fell short in achieving the target of reducing 75% maternal mortality between 1990–2015 [1]. The recent Sustainable Development Goals’(SDGs) target of reducing maternal mortality to less than 70 deaths per 100,000 live births between 2015–2030 may also fall short until attendance and access to skilled antenatal care is improved [6].

World Health Organization (WHO) took an initiative to reduce maternal and newborn mortality by providing greater access to maternal and neonatal care through Emergency Obstetric and Newborn Care (EmONC) program worldwide [7]. This initiative is mainly focused on low and middle-income countries [8]. EmONC refers to ‘care provided in health facilities to treat direct obstetric emergencies that cause the vast majority of maternal deaths during pregnancy, at delivery and during the postpartum period.’[9, p. 193]. Basic EmONC services include “1) administration of parenteral antibiotics to prevent puerperal infection or treat abortion complications; 2) administration of parenteral anticonvulsants for treatment of eclampsia and preeclampsia; 3) administration of parenteral uterotonic drugs for postpartum hemorrhage; 4) manual removal of the placenta; 5) assisted vaginal delivery (vacuum extractions); 6) removal of retained products of conception; and 7) neonatal resuscitation” [10].

Though the initiatives by international organizations have improved maternal and neonatal care globally, some parts of the world still face high mortality in these healthcare domains. For example; despite the launching of EmONC in Pakistan, maternal mortality rate was 180 per 100,000 live births in 2017–18 [11]. Although the rate decreased from 204 in 2010–11 to 180 in 2017–18, it is still among the highest in the world. Pakistan’s neonatal mortality rate, in 2017–18, was 42/1,000 live births [12, 13]. Pakistan is one of the countries that remained unable to achieve 2015 MDGs in major areas like maternal and neonatal care, ranking third in high-mortality countries [14]. It is due to the fact that only 52% of Pakistani births take place in the presence of skilled professionals, and 48% of women go on to have home deliveries. [15]. The major reasons behind this situation are told as; mother’s education, poverty, rural-urban differences in the availability of neonatal care etc. [16]. However, it is surprising that in the presence of obstetric and neonatal care (EmONC) program, Pakistan is facing high maternal and neonatal mortality rates. It raises an obvious question on the implementation of EmONC in the country.

Implementation of EmONC seems a common issue in developing countries with similar maternal mortality rates. For example, Chi, Bulage, Urdal and Sundby found that the “shortage of qualified staff; lack of essential installations, supplies and medications; increasing workload, burnout and turnover; and poor data collection and monitoring systems” were among the common barriers in the delivery of EmONC in Burundi and Northern Uganda [17, p. 1]. In another study, Prata, Passano, Sreenivas, and Gerdts indicated that in Sri Lanka, Malaysia and Honduras the interventions to improve maternal and neonatal care face the challenges like “the availability of unreliable data and the shortage in human and financial resources, as well as limited political commitment” [18]. Hospitals in Rawanda are also facing such problems [19, 20]. Overall, maternal and neonatal care programs are prone to implementation issues in low resource settings [21].

Rural regions in Pakistan can be considered as low resource settings where maternal and neonatal mortality remains high despite the introduction of EmONC in public health facilities of many rural districts. Bahawalpur division is the poorest and mainly rural region where women literacy is very low [22, 23]. In this division, only 42.3% of pregnant women are attended by skilled health professionals. This percentage is much lesser than the province’s average (52.5%) [16]. More than 55% of women are vulnerable to the practices of traditional birth attendants, and 57.5% of deliveries occur at home. Only 10.4% of deliveries are carried out at public sector health facilities, and about 31% deliveries take place at private hospitals [24], out of which 19% are performed at cesarean sections [25]. In Bahawalpur division, 36.2% of pregnant women do not perform even a single antenatal care visit. This percentage is quite high when we compare with other districts such as Rawalpindi (8.6%). Similarly, the percentage of complete four visits is much lower in Bahawalpur division (27.3%) when compared with Rawalpindi (70.4%) [24]. Low antenatal care attendance is causing greater maternal and neonatal mortality which can be reduced by improving EmONC services [26].

In Bahawalpur division, Bahawalnagar is a more deprived rural district. In Bahawalnagar district, EmONC was launched in September 2014 at public health facilities. Unfortunately, most health facilities in the district are not meeting EmONC standards and targets. It may be due to the lack of real and effective implementation of EmONC services in health units even when the pregnant women recognize the need for skilled attendance, and approach to these facilities for such care [27]. This problem is evident from the fact that in March 2016, the executive district officer (EDO) (health) demanded an explanation from the district authorities including the district coordinator of Integrated Reproductive Maternal Newborn Child Health and Nutrition (IRMNCH & N) Program, social organizer IRMNCH; and deputy district officers of Haroonabad and Bahawalnagar regarding the recorded fake deliveries, indicated and highlighted by the chief minister and his road map team [28]. These facts indicate that the program implementation is facing some serious problems.

Nevertheless, little attention has been paid to identify the barriers to the implementation of EmONC in this rural region of Pakistan. Taking into account the possibility of implementation deficiencies, we believe that there is need to identify barriers to the implementation of EmONC. In this regard, meetings were held with the provincial coordinators of IRMNCH & N Program from 18 districts of Punjab, including the district officer health, Bahawalnagar; district coordinator, IRMNCH & N Program; and deputy district officer health, Tehsil Chishtian of Bahawalnagar. These officers confirmed the need for identifying barriers to EmONC implementation in district Bahawalnagar.

Besides identifying implementation barriers, it is important to know the relative importance of these barriers so that the most important areas could be focused [29]. It is important especially in low resource settings where sufficient funds are not available for addressing all issues simultaneously. Pakistan’s health system is facing not only the governance issues but also severe challenges of finance [30]. Recognizing relative importance of each barrier may help policy makers to allocate limited resources to the most important issues.

From the above discussion two formal questions arise. First, what are the interpersonal, organizational, and system-level barriers in implementing basic EmONC services at health facilities of district Bahawalnagar? Second, what is relative importance of each barrier following from the first question? The objective of this study was to uncover interpersonal, organizational, and system level barriers to implementation of basic EmONC services at health facilities of district Bahawalnagar, and determine relative importance of each barrier. We believe that without identifying and prioritizing these barriers, it is impossible to introduce necessary changes for effective implementation of this and other similar programs. The findings of this study may facilitate local authorities and international organizations (such as the UN, WHO, and UNICEF) in improving the quality of maternal and neonatal care by addressing the barriers identified in this research.

## Materials and methods

### Study settings

As already mentioned, this study was conducted in the health facilities of district Bahawalnagar. The public health facilities of this district include 105 basic health units (BHUs) with 204 beds, 10 rural health centers (RHCs) with 200 beds, 10 hospitals with 701 beds including district headquarter (DHQ) and tehsil headquarter (THQ) hospitals [31]. District Bahawalnagar covers an area of 8,878 sq km, with a population of 2,981,219 [32]. Bahawalnagar’s maternal mortality rate was 124/1,000 live births in 2011 [33]. The neonatal mortality rate of this district in 2017–18 was 43/1,000 births [11].

### Research design and sample

This study used sequential exploratory design as indicated in mixed methods appraisal tool (MMAT) version 2011 [34]. In sequential exploratory designs, “the qualitative findings inform the quantitative data collection, and the quantitative results allow a generalization of the qualitative findings” [34]. This design suited well the objective of our study as we intended to identify barriers to EmONC in a qualitative study, and determined the relative importance of these barriers in a quantitative study. In qualitative study, we interviewed the participants. The quantitative study performeda rank order survey of the same key informants.

Data were derived from interviews of key informants. Participants were health service providers involved in providing 24-hour basic EmONC services in the basic health units of district Bahawalnagar. Sample was selected purposively by targeting the subjects with knowledge and expertise about the delivery of EmONC services in the district. An examination of documents at the district coordinator’s office and a discussion with district health officer informed that executive district officer (EDO) health, deputy district officers (health), provincial and district coordinators (IRMNCH & N), in-charges, lady health visitors and midwives (from those health units that fall short in meeting EmONC targets) could be the key informants of this study. Before initiating the interview process, we identified 86 key informants, out of which 79 were available for interview (one executive district officer (EDO) health; two deputy district officers (health); one provincial and one district coordinator (IRMNCH & N): 14 in-charges; 54 LHVs; six midwives). Of the 79 participants, 19 were male (24%) and 60 (76%) were female. The mean age of participants was 32 years, and the mean experience was 7 years. 60 participants (76%) were skilled birth attendants dealing and handling normal delivery patients and 14 (18%) were in-charges/facilitators and 5 (6%) were managers.

Before initiating interviews, an informed consent of the participants was obtained. The respondents’ participation in the study was voluntary. They were assured that their data would remain confidential, and that they would face no negative consequences as a result of participating in this study. Moreover, a documented review and approval from the Ethical Committee for Scientific Research of COMSATS, Vehari Campus, was also obtained for this study.

### Research instrument and interview process

A ‘Key Informant Interview Guide’ was prepared as a major research instrument for collecting data from interviews. This guide has been provided as a supplementary material. Besides its other useful features, this guide provides key topics for interview, ideas to explore, and probes (see Table A in S1 File). The major question was: what are the interpersonal, organizational, and system-level barriers in implementing basic EmONC services in the health facilities of District Bahawalnagar? Comprehensive literature review and a pilot study was performed to refine sub-questions and other components of the Key Informant Interview Guide. This guide was finalized after discussion with two departmental officials and two academic researchers.

For conducting interviews, participants were approached at their concerned health facilities. The time for each interview was 75 to 90 minutes. Before initiating interviews, the participants were informed about the study objectives. An experienced health department official was present for making arrangements to conduct interviews. A research team member was trained to facilitate discussions on issues related to achieving EmONC standards. All the discussions were recorded on flip charts, with the important issues and prominent factors highlighted. For feedback and reconfirmation, the discussion was recalled with the participant highlighting important areas (for ambiguous answers specifically) in the closing notes of the interview. Data collection was completed in four months (two visits: one for interviews and one for obtaining rankings to determine relative importance of identified barriers) including a one-month LHV training break, when interviews were suspended.

#### Rank order survey

In low resource settings, it is important to know the relative importance of problems so that the most important issues could be focused on [29]. The participants who help identify factors or barriers in qualitative survey can better inform about the relative importance of these factors [35]. So, after completing the category search and listing the final barriers, we once again approached to the same respondents who participated in the qualitative survey, and asked them to rank the identified barriers based on their relative importance. A questionnaire, with a list of identified barriers, was presented to the respondents who were asked to allocate a unique number to each barrier depending upon the priority of its importance. This survey provided quantitative data of barriers ranked from the most important to least important.

### Data Analysis

Using data from key informants’ interviews, a qualitative content analysis was performed to identify barriers to implementing EmONC. Subsequently, a quantitative analysis (summed-rank orders) was performed to determine the relative importance of the barriers identified in the content analysis.

#### Qualitative content analysis

Content analysis is a research technique that follows a pattern of components to develop a useful context of valid inferences and replication of text [36, 37]. Content analysis was performed by using insights from previous research [36–38]. Flip charts were used to note the information that emerged from interviews. The collected data were assembled in Microsoft Excel sheet. Accuracy and consistency were ensured by counterchecking the transcribed notes with the flip chart notes. The coding, reducing, and inferring of data were facilitated by ATLAS.ti software. ATLAS.ti is reliable and speedy software that facilitates the analysis of extensive data sets and enables the researchers to rethink and recollect information [39]. ATLAS.ti is used to determine the co-occurrence of an event or its antecedents, and to determine the frequency of codified responses or events [40].

The above process helped categorize the barriers identified from discussions with the participants. It followed a three-step process. The first step involved developing general themes using single-word descriptors that were idiosyncratic in nature. The second step was to test the objectivity and consistency of themes and categories. The third step involved entering the categories in ATLAS.ti, using wild cards to search for specific categories compiled after judgment analysis. Once the wild cards or themes were allotted to a category, searching for a wild card or expression provided all the related paths. The quotations found from the wild card search are called “category hits” [41]. Once the category hits were generated, the paragraphs were reviewed to avoid misplacement and repetition. The misplaced categories were removed from final consideration. The results gathered from themes and networks were then interpreted according to the research question.

Following the steps of content analysis, general themes were generated. Categories, defined in one or two words, were determined after identifying all idiosyncratic interpretations. This determination helped verify the appropriateness of the central question. The next step was to check the objectivity and consistency of the categories through the sample judgment process. Three samples were taken into account through a random sample generator. The sample response was categorized by two independent judges (researchers). The judges provided a number of words that could explain a category. The categories and their definitions were then generated by following the above mentioned steps at all levels.

#### Quantitative analysis (summed-rank orders)

A quantitative analysis was performed on the data obtained from rank order survey. Following Pullig et al., summed-rank orders were calculated to determine the relative importance of each barrier [35]. According to Pullig et al., “summed rank order is calculated as follows: Σ (Frequency × Rank) for each factor. The total lowest score results in the highest-ranking, while the highest total score results in the lowest ranking”. The summed ranks were further analyzed for overall rank differences among various barriers in a group [35]. For this purpose, a non-parametric test, Kendall’s W or Kendall’s coefficient of concordance, was applied.

## Results

### Results of content analysis

The final categories and their analogous definitions for interpersonal, organizational and system-level barriers have been shown in Table 1. The content analysis resulted in 22 categories (seven, eight and seven categories of interpersonal, organizational and system-level barriers, respectively). All the categories emerged from the data gathered for this study. However, some categories are pre-established in the literature and some are specific to this study. For example; in case of interpersonal barriers, lack of interpersonal communication, lack of teamwork, coalition building issues, and interpersonal conflicts were pre-established in literature. The respondents of this study also considered these issues as barriers to EmONC implementation in district Bahawalnagar. The categories like improper use of power and accountability procedures are more specific to this study. Similarly, organizational culture, organizational change, role clarity, lack of leadership, lack of organizational integration, and lack of job security were pre-established organizational level categories. At system-level, the issues of infrastructure, resources, and dual practice were pre-established.

**Table 1:** Categories of interpersonal, organizational and system-level barriers.

**Interpersonal barriers**	**Definition**
Lack of teamwork	Lack of collaboration | lack of cooperation | lack of teamwork
Interpersonal communication	Idea sharing | persuasion | communication
Lack of coalition building	Obstructive alliance | partnership negligence
Improper use of power	Power struggle | deception| unfair use of power | blackmailing | flattery
Interpersonal conflicts	Value system disparity | disproportionate workloads | lack of trust | individual differences
Intra-departmental communication	Feedback concealment | communication gap
Accountability procedure	Absence of responsibility mechanism |prolonged answerability procedure
Lack of training	Training deficiency | improper induction training | lack of up gradation
Lack of leadership	Motivation paucity | lack of ownership
Organizational culture	Uncertain standard operating procedures | organizational citizenship behavior dilemma | absence of value system | corruption
Human resource deployment	Unnecessary general duties | unavailability of staff
Lack of organizational integration	Lack of Inter-organizational relationship | lack of cooperation | secondary-level patient mishandling | negative perception
Job insecurity	Lack of commitment | demotivation | job insecurity | uncertainty regarding the future
Role clarity	Role ambiguity | role incompatibility | lack of information
Organizational change	Rapid change | policy instability | dynamic targets
**System-level barriers**	**Definition**
House-job requirement	On-the-job training | skill development program | science and art integration
Obstacles to disseminating health knowledge	General public knowledge | outdated beliefs | illiteracy
Lack of infrastructure	Lack of health facility protocols | basic framework deficiency | communication and transport
Dual practice	Private practitioners | goal alignment issues | traditional birth attendant services
Resource availability	Stock limitation (medicine) | inadequate medical equipment | financial capital deficiency
LHV knowledge	Capacity building deficiency | knowledge deficiency | quiescent training
High targets	Estimated targets | inappropriate distribution | imbalanced planning

Table 2 shows the frequencies of codes for interpersonal, organizational and system-level barriers. In case of interpersonal barriers, a total of 282 hits were identified. *Lack of teamwork* and *lack of coalition building* were the most discussed categories, resulting in 17% hits (each category). These were followed by *interpersonal conflicts* (15%), *improper power distribution* (14%), and *accountability procedure* (13%). *Interpersonal and intra-departmental communication* were discussed least (12%).

**Table 2:** Frequencies of codes.

Categories	Total Hits	% of total hits per question
**Interpersonal barriers**		
Lack of teamwork	49	17
Interpersonal communication	34	12
Lack of coalition building	47	17
Improper power distribution	39	14
Interpersonal conflicts	42	15
Intra-departmental communication	35	12
Accountability procedure	36	13
Total	282	100
**Organizational barrier**		
Lack of training	27	9
Lack of leadership	39	13
Organizational culture	45	15
Human resource deployment	43	14
Lack of organizational integration	38	12
Job insecurity	47	15
Role clarity	38	12
Organizational change	33	11
Total	310	100
**System-level barrier**		
House job requirement	53	17
Lack in providing health knowledge	37	12
Lack of infrastructure	36	12
Dual practice	41	13
Resource availability	51	17
LHV knowledge	41	13
Higher targets	49	16
Total	308	100

Organizational barriers got 310 hits. *Job insecurity and organizational culture* were the most discussed categories, resulting in 15% hits. In comparison, *lack of training*, at 9%, was discussed least. The other categories that received attention were *human resource deployment* (14%), *lack of leadership* (13%), lack of role clarity (12%), lack of cooperation from secondary health services (12%), and *organizational change* (11%).

The hits for system-level barriers were 308. The most discussed categories were *resource availability* and house-job requirement (17% hits-Table 2). These were followed by *high targets* (16%), *dual practice* (13%), *LHV knowledge* (13%). *Lack of infrastructure* and *lack in providing health knowledge* were discussed least (12%).

### Results of quantitative analysis

As already mentioned, the participants evaluated and ranked the barriers identified in content analysis, and Kendell’s coefficient of concordance was used to examine these ranks.

#### Analysis of interpersonal barriers

Table 3 shows the descriptive statistics. The results indicate that the ranks were remarkably different from each other (Kendall’s W = .0204, chi-square = 86.891, df = 6, p-value < 0.01. Table 4 shows that lack of teamwork and lack of coalition building were ranked first and second, respectively (summed ranks = 244 and 245, respectively). These two factors were not significantly different from each another, indicating that respondents considered them equally important. Also, the next two factors—interpersonal conflicts and interpersonal communication—were not significantly different from one another (summed ranks = 251 and 252, respectively). Interpersonal communication, intra-departmental communication, and accountability procedure were ranked 5^th^, 6^th^, and 7^th^, respectively (summed ranks = 292, 297, and 407, respectively). These factors were significantly different from each other.

**Table 3:** Descriptive statistics (interpersonal barriers).

Kendall’s W for rank differences among interpersonal barriers			
Kendall’s W	Chi-square	Df	Sig.
0.204	86.891	6	.000

**Table 4:** Rank orders (interpersonal barriers).

Interpersonal-Level Barrier Categories	Summed Rank	Percentage Rank (1)	Percent Ranked in Top 2	Percent Ranked in Top 3
Lack of teamwork	244(1)	20	59	68
Lack of coalition building	245(2)	21	30	61
Interpersonal conflicts	251(3)	20	41	56
Improper power distribution	252(4)	1	18	48
Interpersonal communication	292(5)	15	23	30
Intra-departmental communication	297(6)	20	23	25
Accountability procedure	407(7)	3	7	13

Summed rank orders are calculated from highest to lowest: ∑ (Frequency × Ranks) with each factor. The highest score gets the lowest ranking (7) and the lowest score gets the highest ranking (1).

The results indicate that the respondents considered lack of teamwork, lack of coalition building, interpersonal conflicts, and interpersonal communication as the most important interpersonal barriers to basic EmONC services.

#### Analysis of organizational barriers

The results in Table 5 indicate that the ranks of organizational barriers were significantly different from each other (Kendall’s W = .302, chi-square = 149.873, df = 7, p-value < 0.01). Table 6 shows that job insecurity and organizational culture, with significant rank differences, were ranked first and second, respectively (summed ranks = 169 and 204, respectively). The next factors—human resource deployment, role clarity, lack of leadership, and lack of organizational integration—were also significantly different from each other (summed ranks = 266, 342, 365, and 386, respectively). Organizational change and lack of training were ranked 7^th^ and 8^th^, respectively (summed ranks = 391 and 433, respectively). These factors were significantly different from each other. Overall, the results indicate that there is a wide difference between the first three and the rest of organizational barriers. It shows that the respondents considered job insecurity, organizational culture, and human resource deployment as the most important interpersonal barriers to EmONC delivery.

**Table 5:** Descriptive statistics (organizational barriers).

Kendall’s W for rank differences among organizational barriers			
Kendall’s W	Chi-square	df	Sig.
0.302	149.873	7	.000

**Table 6:** Rank orders(organizational barriers).

Organizational-Level Barrier Categories	Summed Rank	Percentage Rank (1)	Percent Ranked in Top 2	Percent Ranked in Top 3
Job insecurity	169(1)	48	69	72
Organizational culture	204(2)	30	52	75
Human resource deployment	266(3)	6	24	54
Role clarity	342(4)	7	11	20
Lack of leadership	365(5)	3	20	27
Lack of organizational integration	386(6)	3	15	23
Organizational change	391(7)	3	7	17
Lack of training	433(8)	1	1	14

Summed rank orders are calculated from highest to lowest: ∑ (Frequency × Ranks) with each factor. The highest score gets the lowest ranking (8) and the lowest score gets the highest ranking (1).

#### Analysis of system-level barriers

Descriptive statistics in Table 7 indicate that there is a significant difference among the ranks of system-level barriers (Kendall’s W = .137, chi-square = 58.376, df = 6, p-value < 0.01). With a significant rank difference from each other, high targets and resource availability were ranked first and second, respectively (Table 8: summed ranks = 206 and 229, respectively). House job requirement and dual practice were ranked as third and fourth, and remained significantly different from each other (summed ranks = 249 and 306, respectively). Lack of providing health knowledge, lack of infrastructure, and LHVs’ knowledge were ranked 5^th^, 6^th^, and 7^th^, respectively (summed ranks = 310, 326, and 362, respectively). These factors were also significantly different from each other.

**Table 7:** Descriptive statistics (system-level barriers).

Kendall’s W for rank differences among system barriers			
Kendall’s W	Chi-square	Df	Sig.
0.137	58.376	6	.000

**Table 8:** Rank orders (system-level barriers).

System-Level Barrier Categories	Summed Rank	Percentage Rank (1)	Percent Ranked in Top 2	Percent Ranked in Top 3
Higher targets	206(1)	24	55	69
Resource availability	229(2)	10	28	63
House job requirement	249(3)	31	51	56
Dual practice	306(4)	8	13	30
Lack in providing health knowledge	310(5)	17	27	31
Lack of infrastructure	326(6)	3	17	32
LHV knowledge	362(7)	7	10	18

Summed rank orders are calculated from highest to lowest: ∑ (Frequency × Ranks) with each factor. The highest score gets the lowest ranking (7) and the lowest score gets the highest ranking (1).

In Table 8, a wide difference was observed between the first three barriers (high targets, resource availability and house job requirement) and rest of the system barriers. It indicates that the first three are the most important barriers to EmONC in the district.

## Quality assessment

The quality of this study was assessed by two independent researchers using mixed methods appraisal tool (MMAT-2011) [34]. Though MMAT was developed for systematic reviews, it can be used for evaluating the methodological quality of individual studies. The MMAT involves a series of questions which are answered by independent reviewers by taking into account the appropriateness of qualitative and quantitative data sources, data analyses procedures, relevance of research design with study objectives, addressing divergence of qualitative and quantitative results (if mixed method is used) etc. Generally, the independent reviewers’ responses are; ‘Yes’, ‘No’, ‘can’t tell’, and ‘comments’. 'Yes' denotes that the quality criterion is fulfilled, 'No' means that the quality criterion is not fulfilled, and 'Can't tell' indicates that the study does not provide sufficient information to determine if the quality criterion is not fulfilled or not [42]. This study’s reviewers ranked ‘yes’ for all questions with some comments which were incorporated by the research team, and re-evaluated by the same reviewers.

## Discussion

The qualitative and quantitative analyses identified various interpersonal, organizational and system-level barriers to EmONC implementation in district Bahawalnagar and determined their relative importance. The most important of these barriers have been discussed below.

### Interpersonal barriers

#### Lack of teamwork

The most important interpersonal barrier to basic EmONC service delivery is teamwork. A team helps members share goals and knowledge, facilitates effective communication, and fosters mutual respect [43–45]. However, the absence or shortage of these factors among coworkers hinders the capacity to cooperate, and leads to poor teamwork. The interviews with care providers informed that coworkers greatly fall short in mutual respect and communication, which are resulting in the absence of goal sharing for implementing EmONC. Though the service providers have the same designations (LHV, midwife), the relationships they share need to be worked on.

The issue of contractual employees (though linked to system and organizational issues) is creating problems at interpersonal level. The LHVs of the IRMNCH& N Program are contractual employees who shoulder the maximum workload owing to their uncertain job structure. Taking advantage of their seniority, the permanent and senior LHVs and midwives try to shift their workload to these contractual employees. Moreover, there is a lack of trust among staff. In some cases, senior staff members do not trust newcomers, believing them to be incompetent. This mistrust leads to seniors restricting their training and not sharing knowledge with juniors. The absence of collaborative effort affects the performance of healthcare providers [45].

#### Lack of conflict management

Respondents ranked lack of conflict management as the second most important barrier. Conflict refers to the difference in opinion, organizational factors, interpersonal relationships, values, and beliefs, causing moral complications [46]. Conflicts are generated when healthcare service providers are unclear about their responsibilities and fail to understand ethical principles [47]. Lack of power, recognition, motivation, and scope for practice and increased workload result in emotional discomfort and conflicts among coworkers [48].

Given the diverse cultural orientations of people serving the health organization, interactional complication are highly likely. Consequently, the enhanced personality and workplace clashes affect the quality of service delivery. Failure to resolve conflicts results in compromising the number and quality of patient care services. This study observed interpersonal conflicts among health service providers. As a result, targets were not met.

#### Interpersonal communication and improper power distribution

Interpersonal communication issues and improper power distribution were ranked 3^rd^ and 4^th^ in the list of important barriers. Interpersonal communication plays vital role in employee job satisfaction through relationship development [49]. Health providers are likely to deliver better healthcare if they are satisfied. Unfortunately, however, health service providers on EmONC program in Bahawalnagar district lack healthy interpersonal communication not only among coworkers but also with program leadership.

Moreover, nurses and LHVs are powerless in decision-making because of their designation and position in the hierarchy [47]. Senior employees wield their power owing to their interpersonal relationships with supervisors and their permanent status. The powerlessness blocks motivation [50] and due to this, powerless employee remain intrinsically excluded from achieving program objective. Consequently, very well-designed programs may fail.

### Organizational barriers

#### Job insecurity

The IRMNCH program hires health service providers (technical and non-technical) on a daily or ad hoc basis with no after-job benefits or job security. Job insecurity leads to uncertain future with lower job commitment and satisfaction as these employees constantly look for more secure employment. Health care employees face distributive injustice in the allocation of legal residences, medical facilities, and economic status imbalance that results in moral discomfort, affecting the quality of service delivery and resulting in the desire to switch jobs [47, 51]. Temporary nature of jobs increases turnover intentions and reduces job commitment. Empirical studies indicate that temporary employees experience less work engagement and job satisfaction than their permanent counterparts (fixed-term employees) [52]. There have been cases where, after receiving training from government health care institutes, temporary employees have switched to private sector, using the acquired skills to earn higher salaries and status in private health care institutions. This phenomenon is among the strongest barriers to implementing EmONC in the district under study.

#### Organizational culture

The culture of an organization reflects the core values that differentiate it from other organizations. Organizational culture can foster innovation that can lead to sustainable development [53]. Studies indicate the clear impact of organizational culture on the quality of public health services delivery. Institutions with weak organizational culture have more turnover, negative work attitudes in service delivery, inconsistent services, increased training costs, and lower productivity [54]. Bahawalnagar’s health department falls short the documented standard operating procedures (SOPs). Value system limitations and lack of organizational citizenship behavior are decreasing job satisfaction and employee morale. Consequently, the implementation of EmONC faces problems.

#### Human resource deployment and role clarity

Human resource deployment is an important barrier in equipping primary health care organizations with skilled birth attendants for 24-hour emergency obstetric care services. The trained LHVs and midwives succeed in getting transferred either to health units in their hometowns or deployed on general duties to RHCs or THQs with no targets. The remaining workforce shares greater workload, causing dissatisfaction and lowering job commitment. This egalitarian distribution of health care service providers on geographic and health facility bases increases employee workloads, delivery waiting times, demotivation, and turnover intentions, reduces the time per patient; and results in poor service delivery [55].

Moreover, health providers of basic EmONC services are not very clear about their roles and responsibilities. Role clarity reduces job-related tension and increases job satisfaction [56]. Role ambiguity, on the contrary, increases workplace tension and professional incompetency. The workplace issues arising from the absence of role clarity are creating problems for EmONC implementation in the district.

### System-level barriers

#### Higher targets: Lack of target management

Lack of target management (i.e. irrational and very high targets) has been identified as the first system-level barrier to EmONC implementation. Unfortunately, similar targets are assigned to low and high resource health units. It creates discomfort among the providers of low resource units. Moreover, achieving targets does not mean that the performance and quality are as per standards. In fact, most employees bypass ethical standards for achieving targets… Target pressure has led to recording fake deliveries, and created other administrative and judicial issues in the delivery system of district Bahawalnagar. It further disturbed the delivery of EmONC.

#### Resource availability

The second important system-level issue is the availability of resources. These resources include a combination of medicine stock, medical equipment, human resource allocation, and financial capital. Owing to the scarcity of health funds, resource allocation in the health facilities of developing countries is a challenge [57]. In district Bahawalnagar, the basic EmONC program has not provided the health units with necessary medical equipment. Each interviewee requested program leaders to avail ultrasound machines and tocometers (for fetal heartbeat and uterine contraction measurement). Availability of medical equipment helps identify pre-mature complexities of normal deliveries. In many health facilities of the district; there is shortage of essential lifesaving medicine. Some health facility in-charges manage to avail medicine at their own personal cost or from the “purchi” fee. Other health facilities and health service professionals avoid taking the risk of attending the deliveries because of resource shortage. The lack of equal resource allocation also results in the development of uncertainty and conflicts among health units and health service providers [47].

#### House-job requirement and dual practice

House-job requirement was removed from the LHV training program for nearly two years. The LHVs who graduated during that period are less experienced and try to avoid handling delivery patients because they lack of confidence. Health units with such LHVs remain reluctant to receive obstetric emergencies even if they have the necessary resources. In addition, there is an issue of dual practice which stems from low salaries in public health system, and government’s open permission for private practice due to the shortage of health providers in the country. The issue of dual practice perpetuates many other problems such as health provider’s lack of attendance at public health facility, lesser intention to attend the cases at public health facility etc. It reduces involvement in their job at public health units. Studies indicate that lower compensation is less harmful than lower job involvement [58]. The struggle of dual practitioners to get patients treated at private health care centers lowers the quality of services at public sector healthcare institutes. This imbalance between public and private practice hinders the achievement of safe health care services for the general public [59].

## Conclusions

Based on data from 79 basic healthcare professionals, this study concludes that EmONC implementation is facing significant interpersonal, organizational, and system-level barriers, and these barriers must be addressed for improving maternal and neonatal care in district Bahawalnagar. This study’s focus on determining the relative importance of identified barriers may help policy makers in addressing these issues. More specifically, we recommend health service providers, managers, policy makers, and international organizations (i.e. WHO, UNICEF etc.) to take steps toward improving teamwork within and across health units, develop an effective conflict management system, provide job security to health providers, and help in establishing a healthy organizational cultures in basic health units of the district. The inability to understand the workforce culture is a management failure and one of the major hurdles in implementing a good healthcare program [60]. The leadership should play an active role in developing workforce harmony for successful execution of the program.

In addition, the targets of health units must be rationalized, house job requirement must be reactivated, and private practice must be discouraged. House job requirement should be complimentary to healthcare service providers and needs an ongoing strategy to equip them with latest knowledge and practical training. The dual practice issue can be addressed by developing policies in favor of public healthcare and the providers be incentivized within this system. Most importantly, the health units must be provided with sufficient resources to implement EmONC program.

### Future research

Although this study is exploratory in nature, and no ‘a priori’ hypotheses were tested, future researchers may determine whether any compelling ‘a posterior’ hypotheses can be developed from exploratory data [61]. Moreover, this study was conducted on a specific healthcare issue in a specific region. Future research may focus on other healthcare issues and other regions.

## Supporting information

S1 Fig (TIFF)

S1 Table Categories for interpersonal level barriers in implementation of EmONC services. (DOCX)

S2 Table Frequency of codes ((interpersonal level issues)). (DOCX)

S3 Table Categories for organizational level barriers in implementation of EmONC services. (DOCX)

S4 Table Frequency of codes (organizational level issues). (DOCX)

S5 Table Categories for system level barriers in implementation of EmONC services. (DOCX)

S6 Table Frequency of codes (system level issues). (DOCX)

S7 Table Descriptive statistics of interpersonal level barriers. (DOCX)

S8 Table Rank orders of interpersonal level barriers. (DOCX)

S9 Table Descriptive statistics of organizational level barriers. (DOCX)

S10 Table Rank order of organizational level barriers. (DOCX)

S11 Table Descriptive statistics of system level barriers. (DOCX)

S12 Table Rank orders of system level barriers. (DOCX)

S1 File Key informant interview guide. (PDF)
