# Supplementary figures and images for: Barriers to implementation of emergency obstetric and neonatal care in rural Pakistan
Source: PLoS One. 2019 Nov 5;14(11):e0224161. doi: 10.1371/journal.pone.0224161 (PMC6830770; doi:10.1371/journal.pone.0224161)

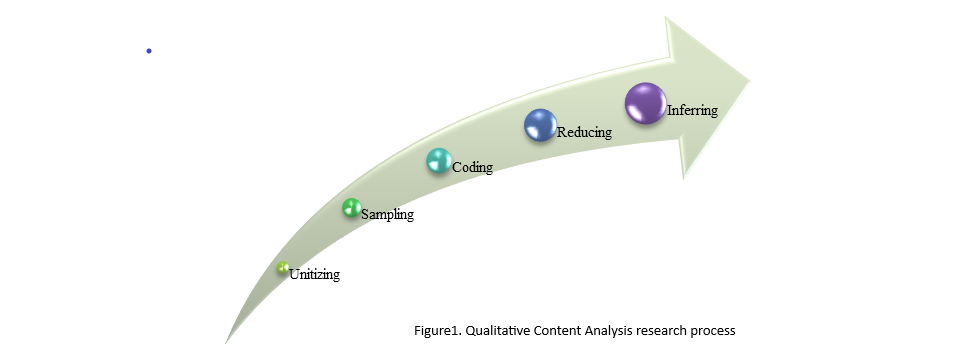

Supplement: S1 Fig — (TIFF) [file pone.0224161.s001.tiff]
