# Supplementary material for: Barriers to implementation of emergency obstetric and neonatal care in rural Pakistan
Source: PLoS One. 2019 Nov 5;14(11):e0224161. doi: 10.1371/journal.pone.0224161 (PMC6830770; doi:10.1371/journal.pone.0224161)
Supplement: S1 Table — (DOCX) [file pone.0224161.s002.docx]

**Table 1. Categories of Interpersonal-Level Barriers in Implementation of EmONC Services**

| Category | Definition |
| --- | --- |
| Lack of teamwork | Lack of collaboration \| lack of cooperation \| lack of teamwork |
| Interpersonal communication | Idea sharing \| persuasion \| communication |
| Lack of coalition building | Obstructive alliance \| partnership negligence |
| Improper use of power | Power struggle \| deception\| unfair use of power \| blackmailing \| flattery |
| Interpersonal conflicts | Value system disparity \| disproportionate workloads \| lack of trust \| individual differences |
| Intra-departmental communication | Feedback concealment \| communication gap |
| Accountability procedure | Absence of responsibility mechanism \|prolonged answerability procedure |
