# Supplementary material for: Barriers to implementation of emergency obstetric and neonatal care in rural Pakistan
Source: PLoS One. 2019 Nov 5;14(11):e0224161. doi: 10.1371/journal.pone.0224161 (PMC6830770; doi:10.1371/journal.pone.0224161)
Supplement: S2 Table — (DOCX) [file pone.0224161.s003.docx]

**Table 2. Frequency of Codes (Interpersonal-Level Issues)**

| What interpersonal-level issues hinder the provision of basic EmONC services? | | |
| --- | --- | --- |
| Interpersonal-Level Barrier Categories | Total Hits | Percentage |
| Lack of teamwork | 49 | 17 |
| Interpersonal communication | 34 | 12 |
| Lack of coalition building | 47 | 17 |
| Improper power distribution | 39 | 14 |
| Interpersonal conflicts | 42 | 15 |
| Intra-departmental communication | 35 | 12 |
| Accountability procedure | 36 | 13 |
| Total | 282 | 100 |
