# Supplementary material for: Barriers to implementation of emergency obstetric and neonatal care in rural Pakistan
Source: PLoS One. 2019 Nov 5;14(11):e0224161. doi: 10.1371/journal.pone.0224161 (PMC6830770; doi:10.1371/journal.pone.0224161)
Supplement: S3 Table — (DOCX) [file pone.0224161.s004.docx]

**Table 3. Categories of Organizational-Level Barriers in the Implementation of EmONC Services**

| What organizational-level issues hinder the provision of basic EmONC services? | |
| --- | --- |
| Category | Definition |
| Lack of training | Training deficiency \| improper induction training \| lack of up gradation |
| Lack of leadership | Motivation paucity \| lack of ownership |
| Organizational culture | Uncertain standard operating procedures \| organizational citizenship behavior dilemma \| absence of value system \| corruption |
| Human resource deployment | Unnecessary general duties \| unavailability of staff |
| Lack of organizational integration | Lack of Inter-organizational relationship \| lack of cooperation \| secondary-level patient mishandling \| negative perception |
| Job insecurity | Lack of commitment \| demotivation \| job insecurity \| uncertainty regarding the future |
| Role clarity | Role ambiguity \| role incompatibility \| lack of information |
| Organizational change | Rapid change \| policy instability \| dynamic targets |
