# Supplementary material for: Barriers to implementation of emergency obstetric and neonatal care in rural Pakistan
Source: PLoS One. 2019 Nov 5;14(11):e0224161. doi: 10.1371/journal.pone.0224161 (PMC6830770; doi:10.1371/journal.pone.0224161)
Supplement: S4 Table — (DOCX) [file pone.0224161.s005.docx]

**Table 4. Frequency of Codes (Organizational-Level Issues)**

| What organizational-level issues hinder the provision of basic EmONC services? | | |
| --- | --- | --- |
| Organizational-Level Barrier Categories | Total Hits | Percentage |
| Lack of training | 27 | 9 |
| Lack of leadership | 39 | 13 |
| Organizational culture | 45 | 15 |
| Human resource deployment | 43 | 14 |
| Lack of organizational integration | 38 | 12 |
| Job insecurity | 47 | 15 |
| Role clarity | 38 | 12 |
| Organizational change | 33 | 11 |
| Total | 310 | 100 |
