# Supplementary material for: Barriers to implementation of emergency obstetric and neonatal care in rural Pakistan
Source: PLoS One. 2019 Nov 5;14(11):e0224161. doi: 10.1371/journal.pone.0224161 (PMC6830770; doi:10.1371/journal.pone.0224161)
Supplement: S5 Table — (DOCX) [file pone.0224161.s006.docx]

**Table 5. Categories of System-Level Barriers in the Implementation of EmONC Services**

| What system-level issues hinder the provision of basic EmONC services? | |
| --- | --- |
| Category | Definition |
| House-job requirement | On-the-job training \| skill development program \| science and art integration |
| Obstacles to disseminating health knowledge | General public knowledge \| outdated beliefs \| illiteracy |
| Lack of infrastructure | Lack of health facility protocols \| basic framework deficiency \| communication and transport issues |
| Dual practice | Private practitioners \| goal alignment issues \| traditional birth attendant services |
| Resource availability | Stock limitation (medicine) \| inadequate medical equipment \| financial capital deficiency |
| LHW knowledge | Capacity building deficiency \| knowledge deficiency \| quiescent training |
| High targets | Estimated targets \| inappropriate distribution \| imbalanced planning |
