# Supplementary material for: Barriers to implementation of emergency obstetric and neonatal care in rural Pakistan
Source: PLoS One. 2019 Nov 5;14(11):e0224161. doi: 10.1371/journal.pone.0224161 (PMC6830770; doi:10.1371/journal.pone.0224161)
Supplement: S6 Table — (DOCX) [file pone.0224161.s007.docx]

**Table 6. Frequency of Codes (System-Level Issues)**

| What system-level issues hinder the provision of basic EmONC services? | | |
| --- | --- | --- |
| System-Level Barrier Categories | Total Hits | Percentage |
| House job requirement | 53 | 17 |
| Lack in providing health knowledge | 37 | 12 |
| Lack of infrastructure | 36 | 12 |
| Dual practice | 41 | 13 |
| Resource availability | 51 | 17 |
| LHW knowledge | 41 | 13 |
| Higher targets | 49 | 16 |
| Total | 308 | 100 |
