# Supplementary material for: Barriers to implementation of emergency obstetric and neonatal care in rural Pakistan
Source: PLoS One. 2019 Nov 5;14(11):e0224161. doi: 10.1371/journal.pone.0224161 (PMC6830770; doi:10.1371/journal.pone.0224161)
Supplement: S8 Table — (DOCX) [file pone.0224161.s009.docx]

**Table 8. Rank orders of Interpersonal-Level Barriers**

| Interpersonal-Level Barrier Categories | Summed Rank | Percentage Rank (1) | Percent Ranked in Top 2 | Percent Ranked in Top 3 |
| --- | --- | --- | --- | --- |
| Lack of teamwork | 244(1) | 20 | 59 | 68 |
| Lack of coalition building | 245(2) | 21 | 30 | 61 |
| Interpersonal conflicts | 251(3) | 20 | 41 | 56 |
| Improper power distribution | 252(4) | 1 | 18 | 48 |
| Interpersonal communication | 292(5) | 15 | 23 | 30 |
| Intra-departmental communication | 297(6) | 20 | 23 | 25 |
| Accountability procedure | 407(7) | 3 | 7 | 13 |

Summed rank orders are calculated from highest to lowest: ∑ (Frequency × Ranks) with each factor. The highest score gets the lowest ranking (7) and the lowest score gets the highest ranking (1).
