# Supplementary material for: Barriers to implementation of emergency obstetric and neonatal care in rural Pakistan
Source: PLoS One. 2019 Nov 5;14(11):e0224161. doi: 10.1371/journal.pone.0224161 (PMC6830770; doi:10.1371/journal.pone.0224161)
Supplement: S9 Table — (DOCX) [file pone.0224161.s010.docx]

**Table 9. Descriptive Statistics of Organizational-Level Barriers**

| Kendall’s W for rank differences among organizational barriers | | | |
| --- | --- | --- | --- |
| Kendall’s W | Chi-square | df | Sig. |
| 0.302 | 149.873 | 7 | .000 |
