# Supplementary material for: Barriers to implementation of emergency obstetric and neonatal care in rural Pakistan
Source: PLoS One. 2019 Nov 5;14(11):e0224161. doi: 10.1371/journal.pone.0224161 (PMC6830770; doi:10.1371/journal.pone.0224161)
Supplement: S10 Table — (DOCX) [file pone.0224161.s011.docx]

**Table 10. Rank Order of Organizational-Level Barriers**

| Organizational-Level Barrier Categories | Summed Rank | Percentage Rank (1) | Percent Ranked in Top 2 | Percent Ranked in Top 3 |
| --- | --- | --- | --- | --- |
| Job insecurity | 169(1) | 48 | 69 | 72 |
| Organizational culture | 204(2) | 30 | 52 | 75 |
| Human resource deployment | 266(3) | 6 | 24 | 54 |
| Role clarity | 342(4) | 7 | 11 | 20 |
| Lack of leadership | 365(5) | 3 | 20 | 27 |
| Lack of organizational integration | 386(6) | 3 | 15 | 23 |
| Organizational change | 391(7) | 3 | 7 | 17 |
| Lack of training | 433(8) | 1 | 1 | 14 |

Summed rank orders are calculated from highest to lowest: ∑ (Frequency × Ranks) with each factor. The highest score gets the lowest ranking (8) and the lowest score gets the highest ranking (1).
