# Supplementary material for: Barriers to implementation of emergency obstetric and neonatal care in rural Pakistan
Source: PLoS One. 2019 Nov 5;14(11):e0224161. doi: 10.1371/journal.pone.0224161 (PMC6830770; doi:10.1371/journal.pone.0224161)
Supplement: S11 Table — (DOCX) [file pone.0224161.s012.docx]

**Table 11. Descriptive Statistics of System-Level Barriers**

| Kendall’s W for rank differences among system barriers | | | |
| --- | --- | --- | --- |
| Kendall’s W | Chi-square | Df | Sig. |
| 0.137 | 58.376 | 6 | .000 |
