# Supplementary material for: Barriers to implementation of emergency obstetric and neonatal care in rural Pakistan
Source: PLoS One. 2019 Nov 5;14(11):e0224161. doi: 10.1371/journal.pone.0224161 (PMC6830770; doi:10.1371/journal.pone.0224161)
Supplement: S12 Table — (DOCX) [file pone.0224161.s013.docx]

**Table 12. Rank Orders of System-Level Barriers**

| System-Level Barrier Categories | Summed Rank | Percentage Rank (1) | Percent Ranked in Top 2 | Percent Ranked in Top 3 |
| --- | --- | --- | --- | --- |
| Higher targets | 206(1) | 24 | 55 | 69 |
| Resource availability | 229(2) | 10 | 28 | 63 |
| House job requirement | 249(3) | 31 | 51 | 56 |
| Dual practice | 306(4) | 8 | 13 | 30 |
| Lack in providing health knowledge | 310(5) | 17 | 27 | 31 |
| Lack of infrastructure | 326(6) | 3 | 17 | 32 |
| LHW knowledge | 362(7) | 7 | 10 | 18 |

Summed rank orders are calculated from highest to lowest: ∑ (Frequency × Ranks) with each factor. The highest score gets the lowest ranking (7) and the lowest score gets the highest ranking (1).
