# Supplementary material for: Barriers to implementation of emergency obstetric and neonatal care in rural Pakistan
Source: PLoS One. 2019 Nov 5;14(11):e0224161. doi: 10.1371/journal.pone.0224161 (PMC6830770; doi:10.1371/journal.pone.0224161)
Supplement: S1 File — (PDF) [file pone.0224161.s014.pdf]

Key Informant Interview Guide

Barriers to implementation of emergency obstetric and neonatal care in rural Pakistan

Target

Target key informants for this study are those healthcare professionals who are directly involved in the implementation of emergency obstetric and neonatal care (EmONC), and have knowledge, experience, professional expertise, and deep understanding of the program. The main categories of key informants are listed below:

- Executive district officer (health)
- Deputy district officers (health)
- Provincial level program coordinator
- District level program coordinator
- The In-charges of the health facility level
- The skilled birth attendants at health facility level (lady health visitors/workers)
- Midwives

Objective

- Identifying interpersonal, organizational and system level barriers to the implementation of emergency obstetric and neonatal care (EmONC) in district Bahawalnagar, Pakistan.

Introduction

- Study introduction, objectives etc.
- Objective of conducting interviews
- Explaining how important the key informants' input is for conducting this study
- Explaining about the confidentiality of information

- Explaining that the participant may skip answering the question for which she/he feels uncomfortable

Background Information

A detailed interview questionnaire is prepared after a literature review. The questionnaire developed has been discussed with academic researchers and program facilitators for refinement. It is therefore recommended to precisely focus on questions developed in open-ended discussion. This will help in simplifying the discussion to be considered in relevant umbrella of question and analysis.

The discussion answers should be considered to develop themes for data analysis. The categories and themes for qualitative data should be developed and their correlation and consistency should be checked.

Assessment Fields

The key informants should discuss the three main categories of barriers to emergency obstetric neonatal care for thematic analysis i.e. interpersonal, organizational and system level. Discuss the questions and add or subtract the questions those are not relevant or highly concerned respectively to develop a batter assessment tool.

Administrative Levels

The three levels of assessments will be mentioned in the introduction section and will be read by the interviewer to the participants. Informed consent will be taken from all the participants.

Terminologies

The semi structured interviews question the participants in oral form. The terms and phrases used in questionnaire backed by theories should be simplified to the interviewees and convenient words should be used to question the insights of interviewees.

Conducting Key Informants Interview

Following points have been considered while conducting interviews. In addition, Table A shows key topics for interview, ideas to explore, and probes.

- ❖ Introduce the program to the participant and take informed consent from the interviewee for that their responses will not be shared with anyone.
- ❖ An experienced health department official will be present during the data collection from health facilities to ensure privacy, for example, making arrangements for separate rooms to conduct the individual interviews.
- ❖ The research team should has trained another person for involvement in the interview process. This person will act as a facilitator during interviews.
- ❖ All the discussions will be recorded on flip charts, with the important issues and prominent factors highlighted.
- ❖ For feedback and reconfirmation, the discussion will be recalled with the participant highlighting important areas (for ambiguous answers specifically) in the closing notes of the interview.
- ❖ The health facility will be revisited for ranking of the barriers to be formulated after data collection.
- ❖ The service providers should be appreciated for their public services and ensured to share results of research if they desire.

Table A: Key topics for interview, ideas to explore, and probes

**Topics**	**Ideas to explore**	**Probe**
Perception about the situation of EmONC in district Bahawalnagar	In your opinion, what is the situation of EmONC in district Bahawalnagar?	Whether EmONC delivery is an issue in district Bahawalnagar ?
Perception about the issues hindering implementation of emergency obstetric and neonatal care	In your opinion, what issues are hindering implementation of EmONC? And, what is their level?	Whether implementation issues are systems level, or interpersonal and organizational level.

Topics	Ideas to explore	Probe
(EmONC) in district Bahawalnagar		
System level barriers that hinder the implementation of EmONC	What system level barriers are discouraging/hindering the delivery of EmONC?	Infrastructure?<br>Availability of resource?<br>Dual practice?<br>Others?
Organizational level barriers that hinder the implementation of EmONC	What organizational level barriers are discouraging/hindering the delivery of EmONC?	Organizational culture?<br>Organizational change?<br>Role clarity issues?<br>Lack of leadership?<br>Lack of organizational integration?<br>Organization does not provide job security?<br>Others?
Interpersonal level barriers that hinder the implementation of EmONC	What interpersonal level barriers are discouraging/hindering the delivery of EmONC?	Lack of interpersonal communication?<br>Lack of teamwork?<br>Coalition building issues?<br>Interpersonal conflicts<br>Others?

Note: This guide has been designed by using insights from following documents:

Mannah, M. T., Warren, C., Kuria, S., & Adegoke, A. A. (2014). Opportunities and challenges in implementing community based skilled birth attendance strategy in Kenya. BMC pregnancy and childbirth, 14(1), 279, 1-12.

Sample Key Informant Interview Guide (Annex 6.5), available at https://www.capacityplus.org/guide-for-applying-the-bottlenecks-and-best-buys-approach.html
